# Production of a Novel Superoxide Dismutase by *Escherichia coli* and *Pichia pastoris* and Analysis of the Thermal Stability of the Enzyme

**DOI:** 10.3389/fnut.2022.850824

**Published:** 2022-03-09

**Authors:** Yang Zhao, Liang Zhao, Weiwei Zhang, Lei Rao, Yongtao Wang, Xiaojun Liao

**Affiliations:** ^1^College of Food Science and Nutritional Engineering, China Agricultural University, Beijing, China; ^2^National Engineering Research Center for Fruit and Vegetable Processing, Beijing, China; ^3^Key Laboratory of Fruit and Vegetable Processing, Ministry of Agriculture and Rural Affairs, Beijing, China; ^4^Beijing Key Laboratory for Food Non-thermal Processing, Beijing, China; ^5^Department of Applied Physics, China Agricultural University, Beijing, China

**Keywords:** superoxide dismutase, chestnut rose, engineering bacteria, thermal stability, molecular dynamics

## Abstract

Previously, a new copper-zinc SOD (CuZnSOD) isolated from chestnut rose (*Rosa roxburghii*) with good stability was described. In this study, the biosynthetic approach was used to create recombinant CuZnSOD. RACE PCR was also used to amplify the full-length CuZnSOD gene from chestnut rose, and the ORF segment was expressed in *E. coli* BL21 and *P. pastoris* GS115. For characterization, the enzyme was isolated in two steps in *E. coli* and one step in *P. pastoris*. The biochemical properties of the two recombinant enzymes were similar, and their optimal reaction pH and temperature were 6.0 and 50°C, respectively. According to molecular dynamics simulation, the CuZnSOD showed high stability from 70 to 90°C, and eight amino acids are important for enzyme thermal stability at high temperatures. This study set the stage for industrial manufacture by filling gaps in the link between conformational changes and the thermal stability of the new CuZnSOD.

## Introduction

Because of its low cost and quick production cycle, synthetic biology is already widely used in industries such as medicine, diagnostics, and food (1). Several microorganisms were chosen as host cells for protein production due to their rapid growth and expression (1, 2). The FDA approved engineered bacteria-produced pegloticase (3) and bovine chymosin (4) for the treatment of chronic gout and food processing, respectively. *E. coli* is widely used as a host cell owing to its high genetic properties and versatile cloning tools (3). And *P. pastoris* is the most widely used eukaryotic expression system in industry due to its capacity to grow at extremely high cell densities on minimal medium and efficient secretory expression (5).

ROS are a class of chemicals and free radicals created by living organisms during aerobic respiration (6). ROS concentration has a significant impact on the physiological and pathological processes of organisms (7). A modest level of ROS contributes to signal transduction in cells, whereas a high level of ROS can damage protein and DNA (8). The body has evolved an antioxidant system to maintain the concentration of ROS, and SOD is the first line of defense against free radicals. SODs are classified into three categories based on their ligand metals: CuZnSOD, FeMnSOD, and NiSOD (9). CuZnSOD is the most important antioxidant enzyme in the SOD family, which is found mostly in eukaryotic cells' cytoplasm, nucleus, peroxisome, and mitochondrial membrane space, as well as bacteria's periplasmic space (10).

SOD has been utilized to treat a variety of disorders caused by reactive oxygen species (ROS) (11). It is an anti-inflammatory medicine that can be used to treat rheumatoid arthritis and improve optic nerve fiber degeneration (12, 13). As a result, SOD has been created into a variety of oral supplements and food additives to boost the body's antioxidant capacity (14). Animal studies revealed that oral SOD supplementation effectively alleviated Type 2 diabetic symptoms (15). Furthermore, as a food additive, SOD effectively decreases the oxygen concentration of liquids (16). High activity SOD juice was produced from chestnut rose (17), and SOD purified from chestnut rose shown greater thermal stability (18, 19) than *Camelus dromedarius* (20) and *Anoxybacillus gonensis* (21). Furthermore, high pressure processing (HPP) can greatly boost SOD activity (19). These characteristics improved the enzyme's potential for use in the food business. However, because there are few publications on plant genome sequences, particularly on the enzyme gene, the enzyme created by bioengineering has not been documented up to date. As a result, it is critical to clone the entire enzyme and find an appropriate expression system.

The full-length gene encoding the CuZnSOD from chestnut rose was cloned in this study. Following that, the expression vectors pET-30a(+)-CuZnSOD and pPIC9K-CuZnSOD for this gene were created, and it was revealed that the gene was expressed heterologously in *E. coli* and *P. pastoris*. Two expression systems were used to isolate recombinant CuZnSOD from chestnut rose, and purification protocols were devised. Following that, the purification products were described and compared. Finally, molecular dynamics methods were utilized to explore how the structure changes at different temperatures, providing a theoretical foundation for future thermal stability advancements.

## Materials and Methods

### Plant Materials and Chemicals

Fresh fruits of chestnut rose (Guinong No.5) were collected from Guizhou province, China. They were promptly frozen in liquid nitrogen and kept at−80°C for total RNA extraction. This study's chemicals and solvents were all of analytical grade.

### Cloning and Expression of CuZnSOD

#### Total RNA Isolation and Reverse Transcription

Total RNA was isolated from the fruits according to the manufacturer's procedure using the RNA Easyspin Isolation kit (Tiangen, China). Its quality and quantity were then assessed using agarose gels (1%, w/v) and a NanoDrop spectrophotometer (Thermo, USA). The reaction was carried out in a thermal cycler (Bio-Rad, China) after first-strand cDNA was generated using an RT kit (Tiangen, China).

#### Rapid Amplification of 5′ and 3′ cDNA Ends

Primers (forward: TCTCCTGGCCTTCATGGTTTCCATAT; reverse: GTAGTCTTGCTAAGTTCATGTCCACC) were designed for amplifying partial CuZnSOD gene from chestnut rose based on conserved sequences of known regions of amino acids in Rosaceae of CuZnSOD (*Potentilla atrosanguinea, Malus pumila, Prunus avium, Prunus mume*) from the NCBI database. The preceding step's first-strand cDNA was employed as a template for the PCR reaction. Then, on a thermal cycler, amplification of unknown genes with these primers yields initial partial clones (Biorad, US). The PCR reaction system is 20 μL: high-fidelity enzyme 10 μL, template 1 μL, water 7 μL, forward and reverse 2 μL. The PCR program was 98°C for 2 min, followed by 30 cycles at 98°C for 10s, 57°C for 25 s, 72°C for 10s, and a final extension of 72°C for 5 min. The products were run on a 1% agarose gel, purified with the DNA Recover Kit (Tiangen, Beijing), and then cloned into the pGM-T vector for DNA sequence analysis with the pGM-T Fast Kit (Tiangen, Beijing).

To acquire full-length clones, a pair of gene-specific primers were developed based on the partial known sequences above. The SMART RACE kit was used to accomplish 3′ RACE and 5′ RACE (Clontech, UA). To identify 5′ upstream transcription start areas and 3′ poly(A) termination signal regions, an initial PCR amplification was done using primers: GSP-F TTGCTCCAGTAGGTGTTACGGGAACT, GSP-R GCCAGCAGGATTGAAGTGTGGTCCAGTT and primer UPM (Universal Primer Mix). The two products were then purified on a gel before being recovered for sequencing.

#### Isolation of Superoxide Dismutase Genes

Using the DNAman program, splice the acquired 3′ and 5′ sequences to generate the full-length CuZnSOD gene sequence. Then, based on the full-length CuZnSOD gene sequence, they created a set of primers, CD-F: CTAATACGACTCACTATAGGGCAAG; CD-R: CTTCAAAGCAAGAATTACTCTATTTATCC, and the first-stand cDNA was utilized as a template for the PCR procedure. The PCR reaction setup was identical to the one described previously. The PCR procedure was 98°C for 2 min, followed by 30 cycles of 98°C for 10 s, 57°C for 25 s, 72°C for 10 s, and a final extension of 5 min at 72°C. The amplified product was then validated on a 1% agarose gel, and the gel was retrieved for sequencing to confirm the spliced gene sequences.

#### Bioinformatics Analysis

The sequence homology was performed with the BLAST program provided by NCBI (https://blast.ncbi.nlm.nih.gov/Blast.cgi). The open-reading (ORF) of nucleotide sequences was analyzed through an online web (https://www.ncbi.nlm.nih.gov/orffinder). Conserved regions were analyzed by the conserved domain database at NCBI. Multiple sequence alignments were performed using DNAman software, and a phylogenetic neighbor-joining (NJ) tree was analyzed using the Mega5 program. Protein properties were assessed through the website tool ProtParam (http://web.expasy.org/protparam/), and the glycosylation sites were predicted using the NetNGlyc tool (http://www.cbs.dtu.dk/services/NetNGlyc/).

#### Construction of Expression Vector pET-30a(+)-CuZnSOD and Expression in *E. coli* BL21

The ORF gene PCR product was cloned in pET-30a(+) to create the expression vector pET-30a(+)-CuZnSOD. To amplify the CuZnSOD ORF, the primers F: GGAATTCCATATGATGGCAAAGGGTGTTGCTGTAC and R: CCGCTCGAGTCCATGGAGACCAATAATACC containing *Nde* I and *Xho* I sites (underlined, respectively) were built using Primer Premier 5.0 (Premier Biosoft, USA). To execute PCR amplification using first-strand cDNA as a template, the system is 20 L and the protocol is 98°C for 2 min, followed by 30 cycles of 98°C for 10s, 63°C for 25 s, and 72°C for 10s. Purified from 1 percent agarose gels digested with *Nde* I and *Xho* I, the PCR product was coupled with the pET-30a(+) expression vector linearized by the two restriction enzymes. The generated vector was transformed into competent *E. coli* DH5α to screen and sequence positive clones of pET-30a(+)-CuZnSOD.

The recombinant plasmid pET-30a(+) was transformed into *E. coli* BL21 (DE3). Then the single positive strain was verified by PCR and inoculated in 3 mL of LB broth containing 0.05 mg/mL of kanamycin. After cells were grown until the OD600 reached about 0.9 at 37°C, isopropyl–d-thio-galactopyranoside (IPTG) was added at a final concentration of 1 mM, and the culture was further grown for 5 h with a rotation speed of 220 rpm at 37°C (22). The cultured cells were collected and lysed by lysozyme for protein expression and solubility analysis. 1 mL of the above-mentioned culture bacteria were collected and transferred to 100 mL of LB for a large-scale culture. The bacteria were harvested at 7,000 × g for 5 min by centrifugation, and then the cell pellets were resuspended in lyse buffer (10 mM Tris-HCl, 100 mM PMSF, pH 8.0) (22). The crude enzyme solution was obtained in the supernatant through two steps of ultrasonication and centrifugation (13,000 × g, 5 min). Then the supernatant and sediment were collected for 15% sodium dodecyl sulfate polyacrylamide gel electrophoresis (SDS-PAGE).

#### Construction of Expression Vector pPIC9k-CuZnSOD and Expression in *P. pastoris* GS115

The targeted fragments of ORF gene were obtained by PCR amplification using primers of F: CCGGAATTCATGGCAAAGGGTGTGGCCGTCCTGTGTTC and R: ATAAGAATGCGGCCGCACCTTGCAGGCCAATAATTCCG with *EcoR* I and *Not* I sites (underlined respectively), respectively. The targeted fragments were designed into the vector pPIC9K, which was an expression vector of *P. pastoris* GS115. These recombinant plasmids were linearized with *Sac*I and electroporated into competent cells of *P. pastoris* GS115.

A single clone was selected from the MD plate and inoculated into 10 mL of BMGY (buffered glycerol-complex medium) medium and incubated until the OD value was 2 at 250 rpm/min, 30°C. The above culture solution is centrifuged at 1,250 × g for 10 min and the supernatant is removed. The cells were harvested and resuspended with 10 mL of BMMY (buffered methanol complex medium). Methanol was added to the culture medium every 12 h to a final concentration of 0.5% for 4 days. Meanwhile, the supernatant was collected by centrifugation at 10,000 × g for 15 min to obtain crude enzyme for protein concentration assay and further purification.

### Characterization of SOD

#### Purification of SOD

After filtering with a 0.22 μm filter membrane, the crude enzyme from *E. coli* was loaded into a 1 mL nickel affinity resin (Bio-Rad, USA). The mixture was incubated at 4°C for 4 h to facilitate recombinant proteins' binding to the column protein. The resin was washed twice with 10 column volumes of binding buffer (5 mM imidazole, 0.5 M NaCl, 20 mM Tris-HCl, pH 7.9) and the non-targeting protein was removed using wash buffer (60 mM imidazole, 0.5 M NaCl, 20 mM Tris-HCl, pH 7.9). The target protein was eluted with 4 column volumes of elution buffer (0.5 M imidazole, 0.5 M NaCl, 20 mM Tris-HCl, pH 7.9). Then the fraction of target protein was desalted using a 5 mL HiTrap desalting column (GE Healthcare, Sweden).

The collected proteins were then purified on an AKTA FPLC system (GE Healthcare, Sweden) using a 24 mL Superdex 200 Increase 10/300 GL column. The column was equilibrated with elution buffer containing 20 mM Tris-HCl, pH 7.9, 150 mM NaCl, 1 mM DTT, 0.5μM Cu^2+^ and 0.5μM Zn^2+^. All purification steps were performed at 4°C (0.5 mL/min, 2 mL fractions), and protein purity and mass were assessed by SDS-PAGE and LC-MS/MS. Then the target protein was stored in protecting buffer (60% glycerol, 20 mM Tris-HCl, pH 7.9) at−80°C for other analyses.

The crude enzyme collected from *P. pastoris* is filtered through a 0.22 μm filter, and the buffer was changed using the GE HiTrap desalting column into the storage buffer (20 mM Tris-HCl, 150 mM NaCl, pH 7.9). The desalted sample is loaded onto a 5 mL Capto Q Sepharose column (GE Healthcare, Sweden) equilibrated with storage buffer, then eluted with a linear gradient of NaCl from 0.15 to 1 mol/L at a flow rate of 0.5 mL/min. The entire purification process taken place at 4°C, and the eluted fractions (4 mL) were collected for protein concentration and activity detection.

#### LC-MS/MS Analysis

Two hundred microliters of purified enzyme with 10 mM DTT were digested, then incubated at 100°C for 5 min. After cooling the solution, it was centrifuged at 14,000 × g for 10 min at 4°C after passing through a 10-kDa filter. The mixed liquid was eluted twice with 200 μL of 50 mM NH_4_HCO_3_, followed by incubation at 37°C for 12 h with 50 μL of lysis buffer (50 mM NH_4_HCO_3_, 1μg pancreatin). After desalting, the samples were separated using an Easy-nLC 1200 (Thermo Fisher Scientific, USA) C18 column.The separation conditions were set as follows: Flow rate: 600 nL/min; injection volume: 0.5 μL; mobile phase A: an aqueous solution of 0.1% formic acid; mobile phase B: 0.1% formic acid and 80% acetonitrile; The elution gradient: 10–13% B for 3 min, 13–29% B for 39 min, 29–37% for 11 min.

The mass analysis of the sample was performed by Orbitrap (Thermo Fisher Scientific, USA) from 350 to 1,500 m/z with an AGC target of 5e5 ions and a maximum injection time of 50 ms. The ionization voltage was set to 2,200 V, and the ion transfer tube temperature was set at 320°C. Tandem MS scan spectra were recorded using higher-energy collisional dissociation with high resolution (15,000). The MS/MS spectra data were searched using Proteome Discoverer software (PD) (version 2.2, thermo Fisher Scientific) against the Universal Protein Resource Database (http://www.uniprot.org) of Novermber 2020.

#### Determination of Enzyme Activity and Protein Content

The SOD activity was detected through a microtiter plate assay method with some modifications using a water-soluble tetrazolium salt (WST-1) (18). Sample solution of 20 μL was added into 200 μL 3 mM water-soluble tetrazolium solution and 20 μL 29 mU xanthine oxidase solution. After incubation at 37°C for 20 min, the absorbance at 450 nm was detected.

Protein content was assayed with a BCA protein concentration kit (Thermo Scientific, USA), using bovine serum protein as a calibration curve standard.

#### Kinetic and Biochemical Characterization of Recombination CuZnSOD

Kinetic parameters for CuZnSOD were determined by pyrogallol assay and experiments for substrate were performed in triplicate using different concentrations from 10 to 100 μM (18). Values for the maximum velocity (V_max_) and half-saturation coefficient (K_m_) were calculated by non-linear regression using Double reciprocal/Lineweaver–Burk plot which was prepared by using the reciprocals of reaction rate (1/V) and substrate concentration (1/S).

Then the purified enzyme was incubated at 70–90°C for 2 h to assay thermal stability. Thermal inactivation kinetics of the enzyme at different temperature was fitted according to the methods reported previously (23) with some modifications, and the half-life of enzyme activity was analyzed through the fitting equation.

The six points to test the pH stability (citrate buffer for pH 4.0 and 5.0, PBS buffer for pH 6.0 and 7.0, Tris-Hcl buffer for pH 8.0 and 9.0) were set. The purified enzyme was incubated at 37°C for 1 h at different pH values to detect the relative activity.

#### Molecular Dynamics Analysis

The initial 3D structure was predicted using the SWISS-MODEL tool (https://swissmodel.expasy.org/) and the sequence was conserved and mapped to the structural surface by the Consurf server (24). Then molecular dynamics simulations were performed using the software Gromacs 2020.4 (25) for 10 ns under the GROMOS 54a7 force field and. The protein was then solvated in a cubic SPC water box with a size of 0.8 Å away from any protein atom and sodium ions were added to neutralize the system. The steepest descent method (ntstep = 5,000) was used to perform energy minimization, and the Particle Mesh Ewald (PME) algorithm was used to deal with long-range electrostatic interactions. Using the Velocity-rescale (V-rescale) thermostat, the system was heated to 27 (300.15 K), 70 (343.15 K), 80 (353.15 K), and 90°C (363.15 K), and MD simulations were run at each temperature for 10 ns.Finally, the Gromacs program was used to calculate the root mean square deviations (RMSD) and root mean square fluctuations (RMSF) of the enzyme at various temperatures.

### Statistical Analysis

All experiments were repeated in triplicate. Microsoft Office 2011 and Origin Pro 2017 (Microcal Software, Inc., USA) were used to analyze the experimental data. The significance was established at the 0.05 level.

## Results and Discussions

### Total RNA Extraction and Cloning of Full Length cDNA

**Figure 1:**
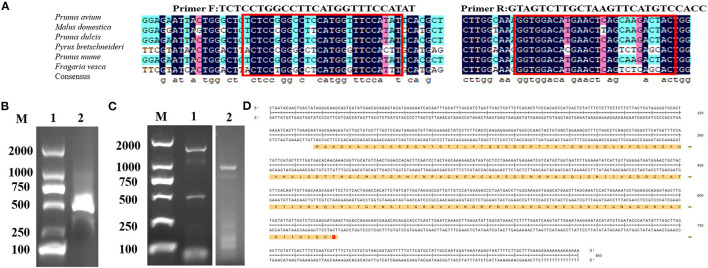
Isolation of full length and molecular cloning of CuZnSOD cDNA **(A)** Multiple-sequence alignment of six nucleo-tides among Rosaceae species were performed using DNAMAN software. Conserved motifs, which were circled in red, were used to design primers for amplifying the unknown internal fragment. **(B)** PCR product of CuZnSOD con-served regions. Lane1: marker DL2000; Lane 2: PCR product analysis on 1% agarose gel. **(C)** Validation of the RACE PCR product of CuZnSOD on 1% agarose gel. Lane 1: marker DL2000; Lane 2: 3′RACE PCR fragment; Lane 3: 5′RACE PCR fragment. **(D)** Deduced amino acid sequence of CuZnSOD from chestnut rose. The full cDNA structure of CuZnSOD from chestnut rose and the ORF region located at position 47–86 contains 459 bp. The deduced amino acid sequences were shown in single-letter code with 498 amino acids.

PCR was performed to get partial genes of CuZnSOD from chestnut rose using primers designed with conserved sequences (Figure 1A) of the Rosaceae family. One clear band at 350 bp was observed on the gels (Figure 1B). Subsequently, the products were recovered to identify the sequence. The sequence alignments were performed by NCBI BLAST. It showed that the specific band was highly identical with the CuZnSOD genes of the Rosaceae family. Hence, the gene sequence can be used to design primers to amplify the cDNA.

Then the gene sequence was used to design gene-specific primers to perform 3' RACE and 5' RACE, and the two programs obtained about 600 bp (3′ RACE product) and about 350 bp (5′ RACE product) specific fragments (Figure 1C), respectively. The two fragments of the RACE reaction were recovered and inserted into the T vector for sequencing. According to the sequencing results and bioinformatics analysis, the full length of the CuZnSOD sequence was 863 bp (Figure 1D), containing ORF 459 bp encoding 153 amino acids.

### Identification and Sequence Analysis of CuZnSOD

**Figure 2:**
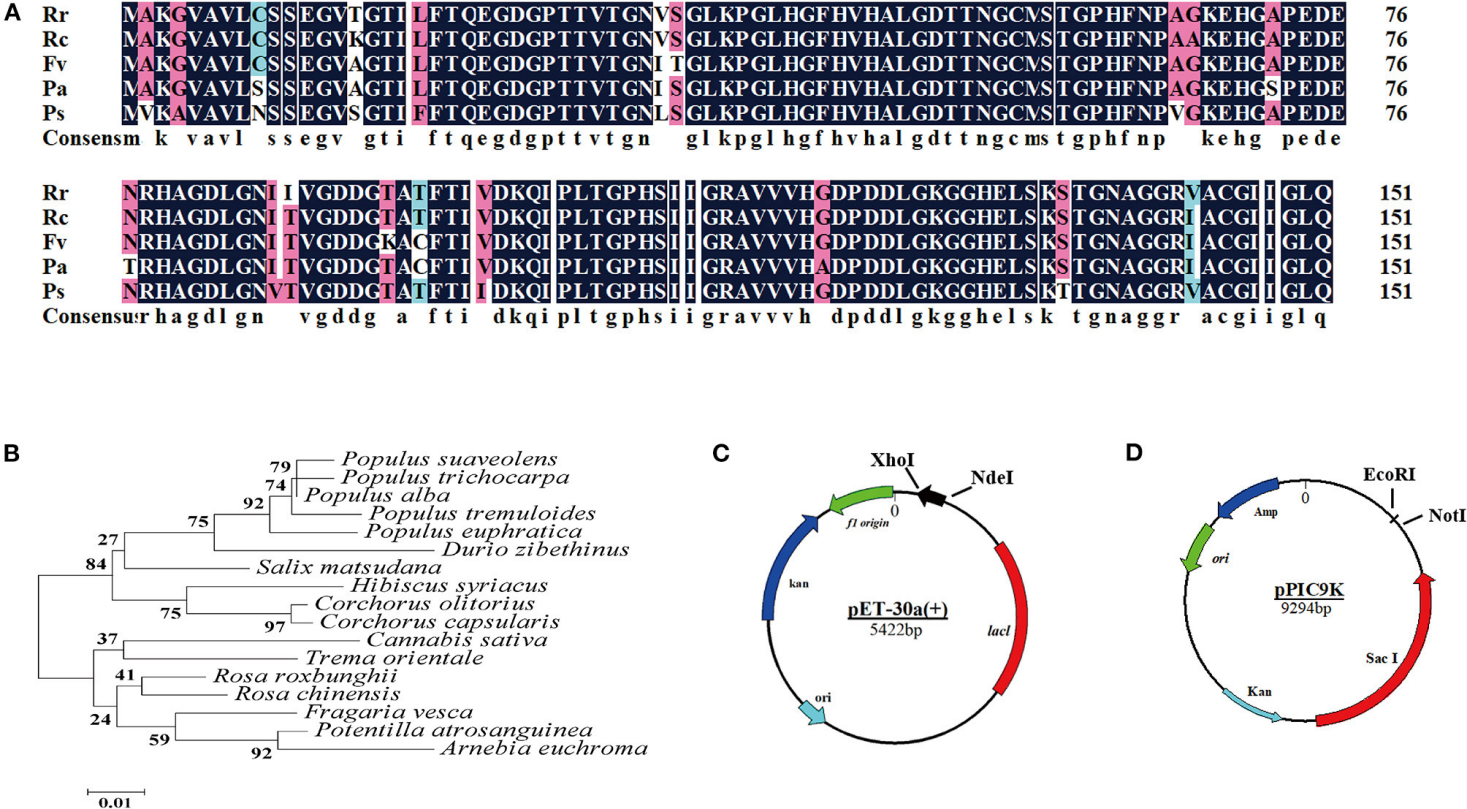
Bioinformatics analysis of CuZnSOD from chestnut rose **(A)** Multiple sequence alignment of chestnut rose CuZnSOD and homologous proteins from other species: Rr (*Rosa roxbunghii*), Rc (*Rose chinensis*), Fv (*Fragaria vesca subsp. vesca*), Pa (*Potentilla atrosanguinea*), Ps (*Populus alba*). Identical residues were shaded in black and similar residues were shaded in cyan and fuchsin; **(B)** Phylogenic analysis of recombinant CuZnSOD with the enzyme from other plants recorded in the NCBI database (https: //blast.ncbi.nlm.nih.gov/Blast.cgi#). The neighbor-joining phylogenic tree was generated using MEGA5, node values represent percentage frequencies of bootstraps generated in 2000 replicates; **(C)** Schematic depicting the construction of expression plasmid pET-30a(+). The CuZnSOD sequence with 6 His-tag was inserted into *Xho* I and *Nde* I site of pET-30a(+) vector multiple cloning site (MCS). The black arrows show the direction of transcription of chestnut rose CuZnSOD gene; **(D)** The structure of pPIC9K and the chestnut rose CuZnSOD was inserted into *EcoR* I and *Not* I.

BLASTp searches revealed that the amino acid sequence of CuZnSOD from chestnut rose has high homology with *Rosa chinensis* (97.37%), *Fragaria vesca subsp. Vesca* (95.39%), *Potentilla atrosanguinea* (94.08%), and *Populus alba* (92.76%). There were different 19 amino acids among these sequences (Figure 2A). Based on the Conserved Domain database (CDD) from NCBI, the active sites of CuZnSOD, which contains the metal ion binding sites, are highly conserved from *Rosaceae*. His 45, His 47, His 62, and His 119 were linked to Cu ligand as one binding site, while His 62, His 70, His 79, and Asp 82 were linked to Zn ligand as the other binding site. The two metal-binding sites constituted an active catalytic site of CuZnSOD.

The Mega 5.0 software was used to draw a phylogenetic tree based on the amino acid sequences of chestnut rose CuZnSOD and the four CuZnSODs. The results (Figure 2B) showed that the protein encoded by the chestnut rose CuZnSOD gene had the closest relationship with CuZnSOD from *R. chinensis*. Therefore, the coding region of CuZnSOD was cloned into the expression vector pET-30a (+) for transforming into *E. coli* BL21 (Figure 2C) and pPIC9K for *P. pastoris* (Figure 2D).

According to the results calculated through the ProtParam website (https://web.expasy.org/protparam/), the molecular weight and isoelectric point of the CuZnSOD were respectively 15.2 kDa and 5.77. From the results, the amino acid sequence of the enzyme contains only one N-glycosylation site (N33).

### Expression and Purification of Recombinant CuZnSOD in *E. coli*

**Figure 3:**
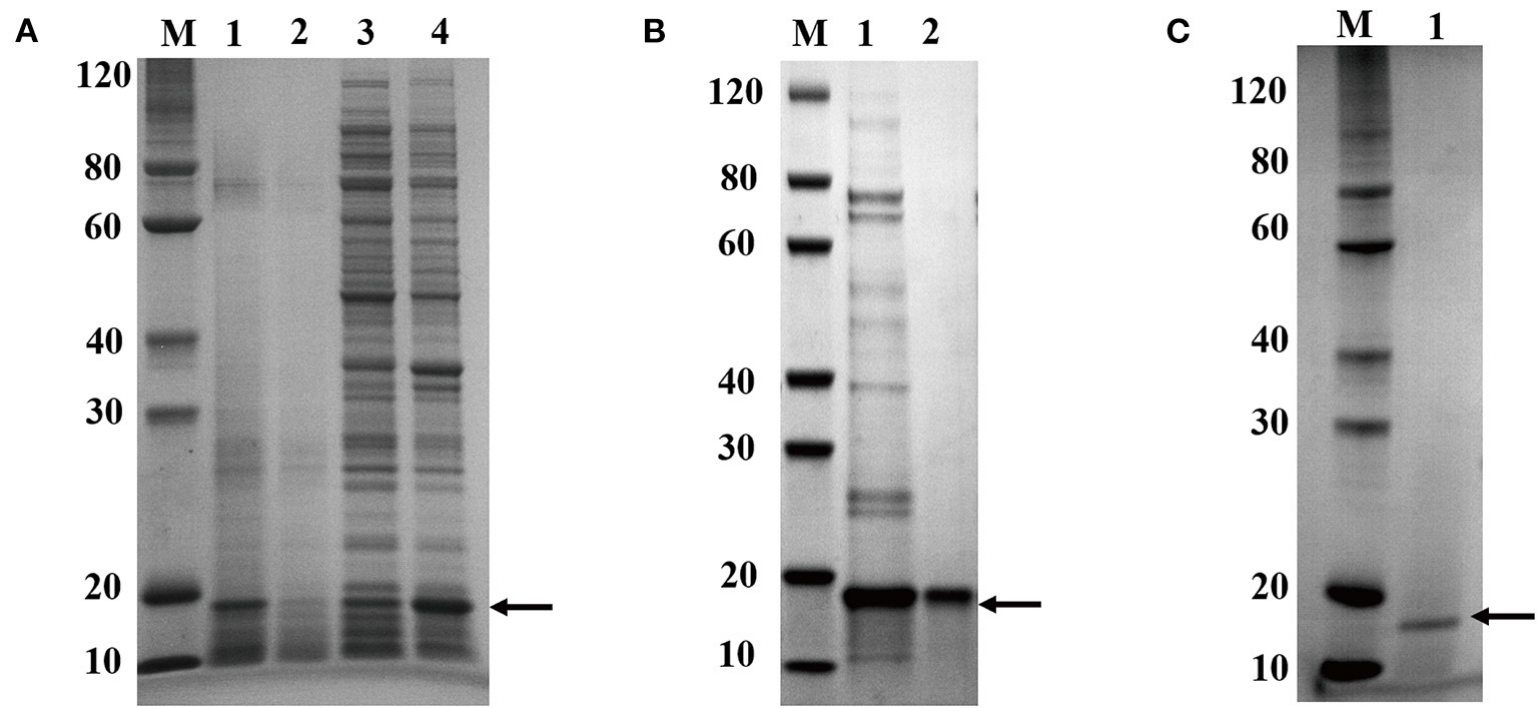
Electrophoresis analysis of recombinant CuZnSOD expression and purification of recombinant CuZnSOD. **(A)** 15% SDS-PAGE analysis of CuZnSOD expression. Lane M: the standard protein marker; Lane 1: total proteins from *E. coli* BL21 with IPTG induction; Lane 2: total proteins from *E. coli* BL21 without IPTG induction; Lane 3: total precipitated proteins from *E. coli* BL21 with IPTG induction after high-speed centrifugation; Lane 4: total superna-tant fluid proteins from *E. coli* BL21 with IPTG induction after high-speed centrifugation; **(B)** 15% SDS-PAGE analysis of CuZnSOD purification. Lane M: the standard protein marker; Lane 1: the proteins eluted from the Ni^2+^-NTA with 500 mM imidazole buffer; Lane 2: peak fraction sample after gel-filtration chromatography purification step; **(C)** 15% SDS-PAGE analysis of CuZnSOD purified from yeast. Lane M: the standard protein marker; Lane 2: SDS-PAGE analysis of the sample after Capto Q Sepharose column purification.

After induction for 5 h, the *E. coli* cells were harvested and broken ultrasonically for electrophoresis. The electrophoresis showed that the recombinant protein was successfully over-expressed in *E. coli* BL21 in the supernatants (Figure 3A). Its protein content and SOD activity were 7.2 mg/mL and 8,200 U/mL, respectively. The proteins in the supernatant solution were preliminarily purified through Ni-IDA metal affinity chromatography (Figure 3B) at a protein content of 0.9 mg/mL, and the SOD activity was 7,748 U/mL. After that, the proteins were further purified by gel filtration (Figure 3B, Supplementary Figure 1A). The final protein content and the SOD activity were 0.45 mg/mL and 6,674 U/mL. Following all the purification steps, the protein was purified by about 13-fold. The SOD specific activity was 14,832 U/mg (Table 1). Due to the two-step effective purification process in this study, the specific activity is higher than one of the SODs from *Anoxybacillus gonensis* and *Aspergillus ficuum* (21, 26).

**Table 1:** Comparison of recombinant SOD produced by different expression systems.

**Expression systems**	**Purification steps**	**Total protein (mg)**	**Total activity (U)**	**Specific activity (U/mg)**	**Purification-fold**	**Yield (%)**
*E. coli* BL21	Crude extractive	72.0	82,000	1,139	1.0	100.0
*E. coli* BL21	Affinity chromatography	3.6	30,993	8,609	7.6	37.8
*E. coli* BL21	Size-exclusion chromatography	0.9	13,349	14,832	13.0	16.3
*P. pastoris* GS115	Crude extractive	49.8	368,150	7,393	1.0	100.0
*P. pastoris* GS115	Ion exchange chromatography	13.2	177,883	13,476	1.8	48.3

After gel filtration, the proteins were analyzed using LC-MS/MS. The coverage of peptides matching the target enzyme was 49%, and the 15 kDa protein was identified as the CuZnSOD gene product by searching in the Uniprot database. The purity of the proteins was calculated as a percentage of abundance in relation to the total sum of abundance, hence the purity of the recombinant CuZnSOD was 99.99% (Supplementary Table 1). The purity (0.99) of the CuZnSOD protein in this study is higher than the 0.95 of CuZnSOD in previous reports (27, 28).

### Expression and Purification of Recombinant CuZnSOD in *P. pastoris*

The target enzyme expressed in yeast was secreted into the supernatant, and the secretion degrades after 60 h, so the sample was collected for analysis before degradation. More importantly, the expression products were analyzed by LC-MS/MS (Supplementary Table 2). It showed that the band is CuZnSOD and its purity is 72.78%. Signal peptides can also drive exogenous proteins produced in *P. pastoris* to the culture medium. This greatly simplifies the recycling of products (29). The crude enzyme solution was purified in one step to reach a purity of over 99% (Figure 3C, Supplementary Figure 1B). Compared with the above-mentioned *E. coli* expression system, less time is required for protein purification of the *P. pastoris* expression system, and it is useful for commercial applications. The content of the enzyme reached 5.0 mg/mL and the 36,815 mL after 60 h of induction (Table 1). The expression level of a thermal SOD (17 kDa) was 1.54 mg/mL after 6 days of incubation for *P. pastoris* (30). The enzyme showed an activity of 13,476 U/mg after purification, with a yield of 48.3% and a 1.8-fold purification (Table 1). Chen et al. (31) used *P. pastoris* to produce human extracellular superoxide dismutase with a yield of 0.4 mg/mL. In this study, the higher yield of recombinant CuZnSOD from chestnut rose may be due to these reactions. First, the ORF gene of chestnut rose CuZnSOD was modified by codon optimization based on yeast bias (32). Second, the increase in expression may be associated with this novel gene. Different genes lead to different protein yields. Third, the higher copy number of the gene in this recombinant strain leads to its increased expression.

### Determination of Kinetic Parameters

**Figure 4:**
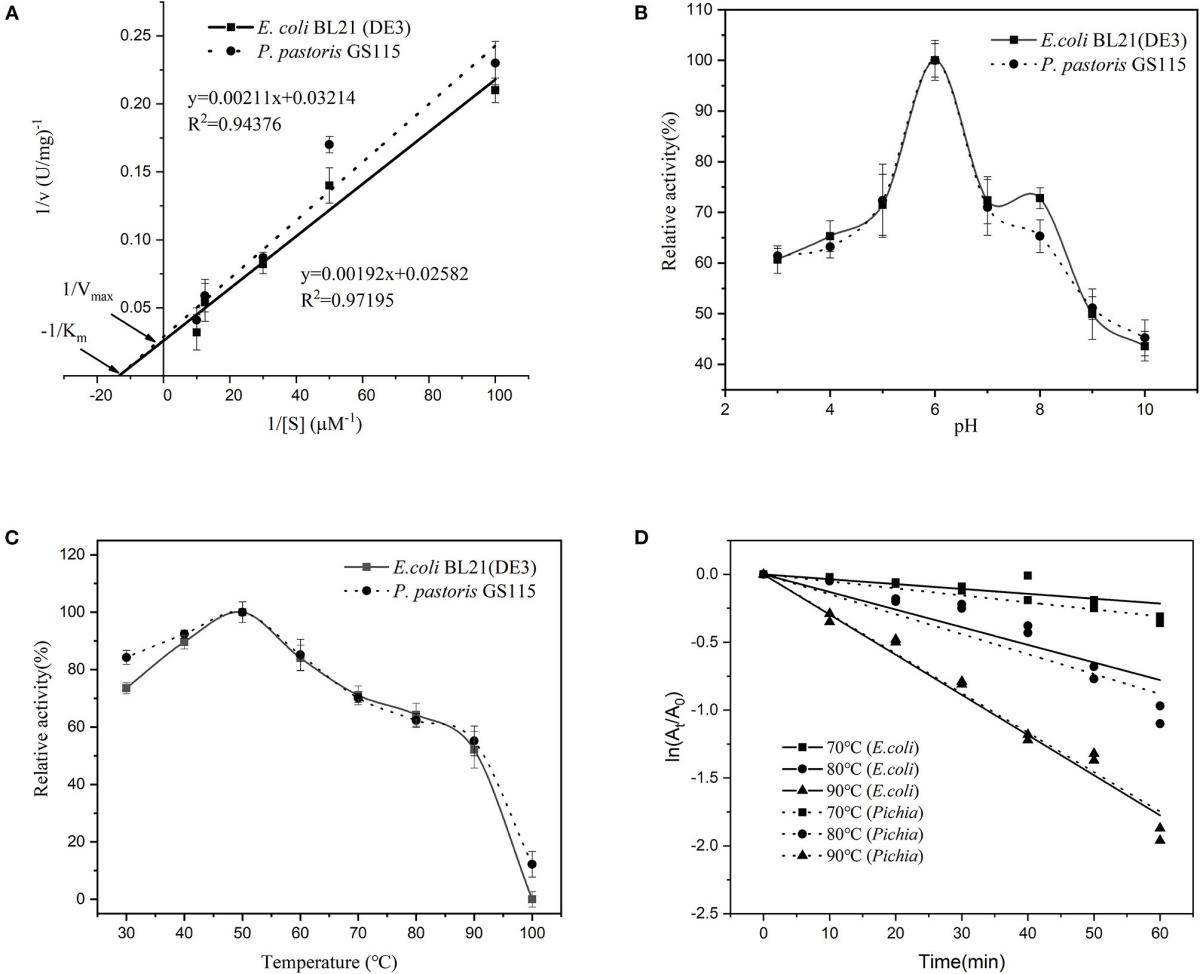
Characterization of recombinant CuZnSOD produced in *E. coli* and *P. pastoris*. **(A)** Kinetic constants of recombinant CuZnSOD; **(B)** Optimal pH of recombinant CuZnSOD. The abscissa indicates the pH of incubation, the ordinate indicates the logarithm of the relative activity; **(C)** The optimal temperature of recombinant CuZnSOD and the maximum activity was set as 100%; **(D)** The kinetics of the thermal inactivation of recombinant CuZnSOD at 70°C (square), 80°C (circular), and 90°C (triangle).

The kinetic parameters of Vmax and Km of the recombinant SOD from *E. coli* and *P. pastoris* were calculated through Lineweaver-Burke plots, which were fitted by linear regressions as evident from *R*^2^ = 0.9720 and *R*^2^ = 0.9438 (Figure 4A). The Km and Vmax values of the recombinant CuZnSOD from *E. coli* were 0.07 mmol/L and 38.73 mmol/min, respectively, whereas the values from *P. pastoris* were 0.07 mmol/L and 31.11 mmol/min. In this study, *E. coli* and *P. pastoris* exhibited the same Vmax and Km values. It has also been previously shown that the enzymes expressed in both systems have the same Km (33). However, it has also been shown that *P. pastoris* expresses enzymes with higher Km values than those of *E. coli*, which is one of the possible reasons the enzyme was glycosylated (34). Vmax correlates with the specific activity of an enzyme. The same Vmax values were measured after dilution in this research.

### Properties of Recombinant CuZnSOD

Both the enzymes showed the same optimum pH and temperature at 6 and 50°C (Figures 4B,C), respectively. Furthermore, the enzyme exhibited high temperature-dependence stability, retaining more than 30% relative activity after 1 h incubation at 70–80°C and about 20% relative activity at 90°C (Figure 4D). It shows that both sources of recombinant enzymes have similar thermal stability. Both recombinant SODs are unstable at high temperatures, particularly at 90°C, when compared to natural SOD, which lost only 16.7% activity after 60 min of incubation at 90°C (18). Our research compared the properties of recombinant enzymes produced by two different expression systems and discovered that they are similar. A similar observation has been reported previously (34, 35), where the recombinant acid protease and β-glucuronidase exhibited similar properties in both *E. coli* and *P. pastoris* expression systems. In addition, the heat stability of recombinant CuZnSOD in this research was exceptional. The enzyme showed a half-life of 50 min at 80°C. The thermal stability of the recombinant enzyme is higher than that of most sources of CuZnSOD. The half-life of a thermostable recombinant CuZnSOD from *Chaetomium thermophilum* in *P. pastoris* is 22 min at 80°C (30). The recombinant CuZnSOD from the jellyfish Cyanea capillata retained 40% activity at 80°C for 10 min, but almost completely lost activity when the incubation time reached 40 min (36); and the engineered SOD from *Anoxybacillus gonensis* lost activity completely at 80°C for 10 min (21). The unique thermostability features make CuZnSOD a great candidate for industrial applications.

### Structural Features of Recombinant CuZnSOD

**Figure 5:**
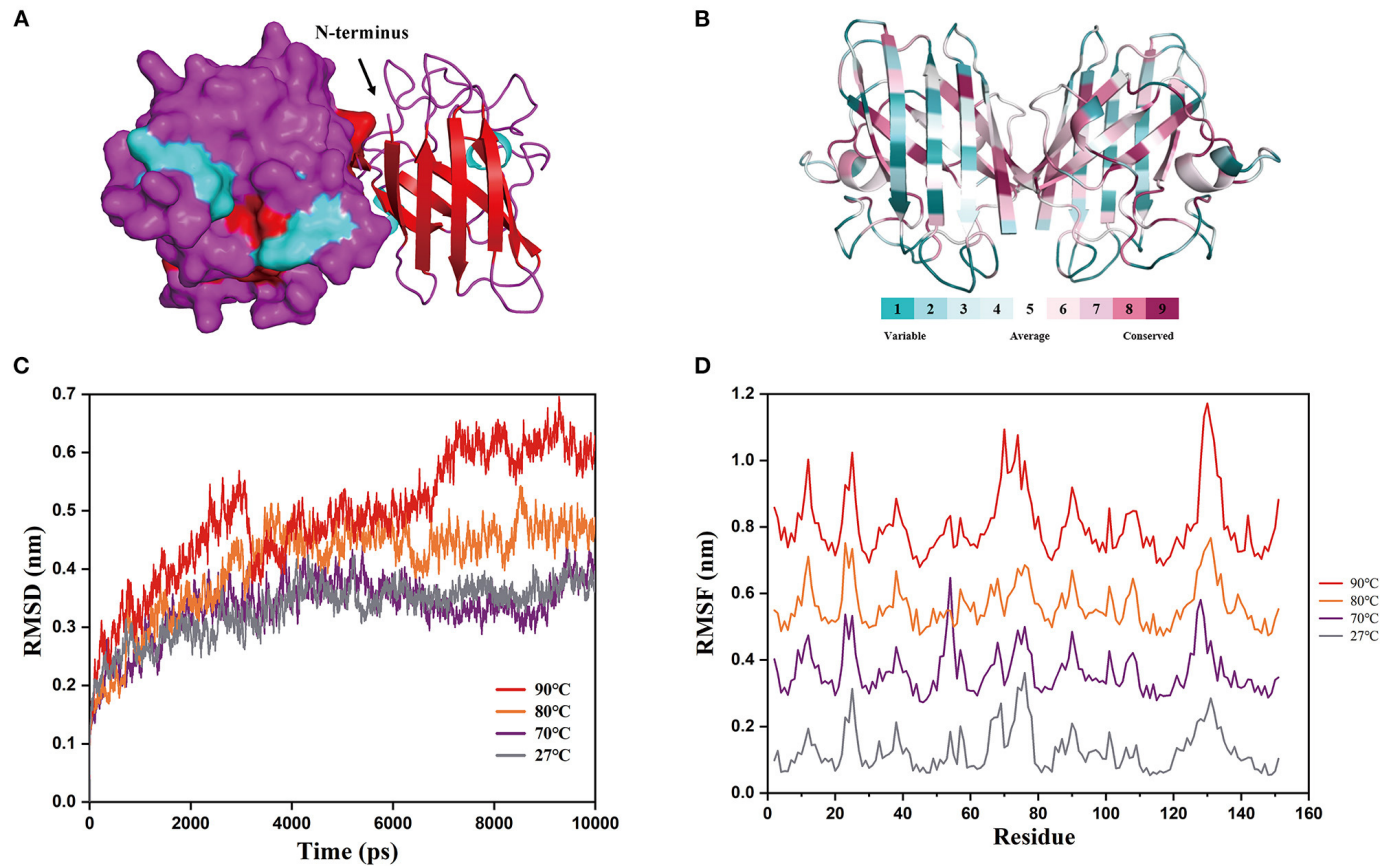
Structural properties and molecular details at various temperatures. **(A)** 3D structure prediction of chestnut rose CuZnSOD. Searching structure templates by using a Swiss-model modeling program, it was found that PDB: 2q2l, 3km2, and 3sod had high identity with the CuZnSOD sequences from chestnut rose. However, only the PDB: 3km2 structure was not artificially modified, so it was used for homology modeling as a template. α-helixs are shown in cyan, β-sheets are shown in red, and random-coil is shown in purple. Electrostatic potential energy and cartoon form are used to represent the left and right subunits, respectively. **(B)** The conservation of the chestnut rose CuZnSOD sequence mapped onto the protein structure. The color of various values represented the protein's surface. A higher value indicated a more conservative sequence, while a lower value indicated a more divergent sequence. **(C)** Root mean square deviations (RMSD) at different temperatures. **(D)** Root mean square fluctuations (RMSF) of each amino acid at different temperatures.

According to the prediction method of homology modeling, the 3D structural model of chestnut rose CuZnSOD was constructed by Swiss-model. As shown in Figure 5A, chestnut rose CuZnSOD is a homodimer consisting of two subunit domains, so its molecular weight in the dimer state was 30.4 kDa. It was similar to the molecular weight of other CuZnSODs from *Potentilla atrosanguinea, Anoxybacillus gonensis*, and camel (20, 21, 37). The active site was made up of a groove with β-sheets and α-helix (Figure 5A). The amino acid in the groove serves as a catalyst by connecting to CuZn (38).

The two subunits are combined by hydrophobic interaction, and the active site is comprised of an imidazole bridge constructed by Cu and Zn ions. Each subunit is composed of 8 β-sheets, which is similar to the enzyme from *Brassica campestris* (39), and three random coils. These eight antiparallel β-sheets form a cylindrical structure, and they are essential for the stability of the enzyme (40). The conserved sequences are found at the active site (Figure 5B), and the non-conserved sequences are found in random coils and the outer three β-sheet sequences. This suggests that the conserved residues are essential for the catalytic activity of the CuZnSOD families (38).

The results of molecular dynamics simulations based on the above structures are shown in Figure 5C. The protein structure reached an equilibrated state at different temperatures according to the RMSD values. The graph shows that the RMSD values at 90°C are much greater than those at the other temperatures. This indicates that the protein structure is refolded at high temperatures (41), resulting in its inactivation. This corresponds to the stability of the enzyme in the above experiment. As determined based on kinetic trajectories to investigate the flexibility of each amino acid residue at different temperatures (Figure 5D), the RMSF values of eight amino acids varied greatly at high temperatures (Residues 12, 25, 70, 74, and 129–132). The residues (12, 25, 70, 74) were in the outermost α-helix, and the remaining residues are in the random coils. It demonstrated that the β-sheets of the enzyme maintained the structure of the protein at high temperatures, and mutating the eight residues will improve the stability of the protein (42).

## Conclusions

In this research, the CuZnSOD from chestnut rose was successfully cloned using RACE and constructed into the pET-30a(+)-CuZnSOD and pPIC9K-CuZnSOD vectors. The gene was then was expressed in *E. coli* BL21 and *P. pastoris* GS115, and two purification procedures were developed to purify the enzyme for further analysis. The enzymes produced by the two systems have biochemical properties that are identical, while yeast is more suitable for industrial production since it can be fermented at high densities. In comparison with known SODs, the enzyme was shown to be more stable over a wide temperature range, making it suitable for commercial applications. Furthermore, the structure was described at various temperatures, laying the groundwork for improving enzyme stability.

## Data Availability Statement

The original contributions presented in the study are included in the article/Supplementary Materials, further inquiries can be directed to the corresponding author/s.

## Author Contributions

YZ, YW, and XL designed the study. YZ and LZ carried out the experiment and performed data analyses. YZ and WZ wrote the article and discussed the results. LR and XL revised the article and provided funding to support the study. All authors contributed to the article and approved the submitted version.

## Funding

This work was supported by the National Natural Science Foundation of China (NSFC) (grant number: 31930087).

## Conflict of Interest

The authors declare that the research was conducted in the absence of any commercial or financial relationships that could be construed as a potential conflict of interest.

## Publisher's Note

All claims expressed in this article are solely those of the authors and do not necessarily represent those of their affiliated organizations, or those of the publisher, the editors and the reviewers. Any product that may be evaluated in this article, or claim that may be made by its manufacturer, is not guaranteed or endorsed by the publisher.
