# Supplementary material for: Production of a Novel Superoxide Dismutase by Escherichia coli and Pichia pastoris and Analysis of the Thermal Stability of the Enzyme
Source: Front Nutr. 2022 Mar 9;9:850824. doi: 10.3389/fnut.2022.850824 (PMC8959677; doi:10.3389/fnut.2022.850824)
Supplement: Supplementary file 1 [file Data_Sheet_1.docx]

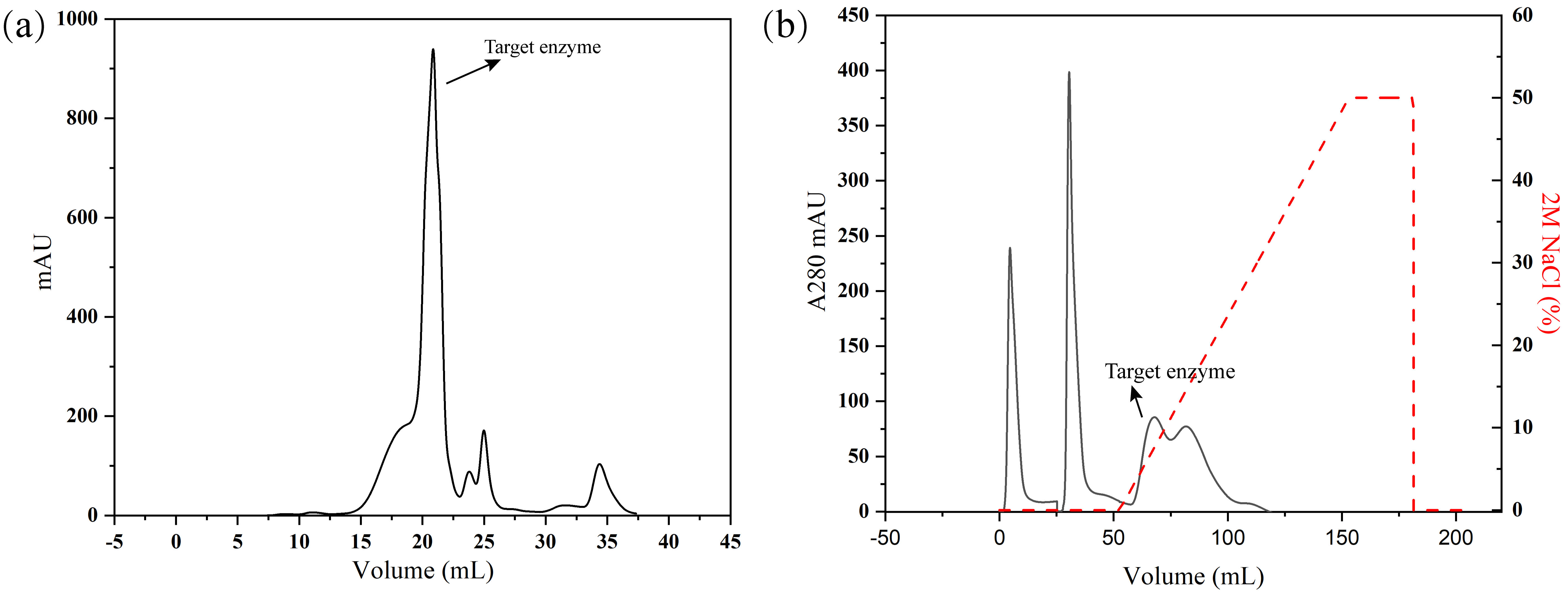


**Supplementary Figure 1.** Chromatogram of recombinant SOD. (a) The Size-exclusion chromatography profile of the recombinant SOD from *E. coli* was eluted with buffer. The components to be collected are indicated by arrows on the peaks. (b) The elution profile of the recombinant SOD from *P. pastoris*. Arrows on the peaks indicate the components to be gathered. The change in NaCl concentration is shown by the red-dash line.

**Table S1**. LC-MS/MS identification of isolated proteins expressed in *E.coli*

| # | Description | Gene name | MW  (kDa) | Abundances |
| --- | --- | --- | --- | --- |
| 1 | Superoxide dismutase [Cu-Zn] | RchiOBHm_Chr3g0452901 | 15.2 | 15786191785 |
| 2 | Maltodextrin-binding protein | malE | 43.4 | 479859 |
| 3 | 50S ribosomal protein | rplL | 12.3 | 189447 |
| 4 | LysR family transcriptional regulator | FAZ83_08530 | 33.4 | 516429 |
| 5 | 6-phospho-beta-glucosidase | chbF | 50.4 | - |
| 6 | 30S ribosomal protein | rpsF | 15.7 | 103573 |
| 7 | Alpha/beta hydrolase | FAZ83_08635 | 33.2 | - |
| 8 | S-(hydroxymethyl) glutathione dehydrogenase | frmA | 39.3 | - |

**Table S2** LC-MS/MS identification of isolated proteins expressed in *P. pastoris*

| # | Description | Gene name | MW  (kDa) | Abundances |
| --- | --- | --- | --- | --- |
| 1 | Superoxide dismutase [Cu-Zn] | RchiOBHm_Chr3g0452901 | 15.2 | 5186800000 |
| 2 | Endo-β-1,3-glucanase involved in incorporation of newly synthesized mannoprotein into cell wall | *BGL2 (K.pastoris)* | 33.7 | 171190000 |
| 3 | Elongation factor Tu | RchiOBHm_Chr6g0291851 | 125.8 | 0 |
| 4 | LysR family transcriptional regulator | FAZ83_08530 | 45.7 | 1149400000 |
| 5 | 6-phospho-beta-glucosidase | chbF | 66.5 | 302830000 |
| 6 | 30S ribosomal protein | rpsF | 187.4 | 0 |
| 7 | Alpha/beta hydrolase | FAZ83_08635 | 71.871 | 286710000 |
| 8 | S-(hydroxymethyl) glutathione dehydrogenase | frmA | 105.47 | 29923000 |
